# BMAL1 collaborates with CLOCK to directly promote DNA double-strand break repair and tumor chemoresistance

**DOI:** 10.1038/s41388-023-02603-y

**Published:** 2023-02-02

**Authors:** Canfeng Zhang, Liping Chen, Lu Sun, Heping Jin, Kai Ren, Shiqi Liu, Yongyu Qian, Shupeng Li, Fangping Li, Chengming Zhu, Yong Zhao, Haiying Liu, Yan Liu

**Affiliations:** 1 The Seventh Affiliated Hospital, Sun Yat-Sen University, Shenzhen, 518107 China; 2 The Center for Medical Research, The First People’s Hospital of Nanning City, Nanning, 530021 China; 3 MOE Key Laboratory of Gene Function and Regulation, School of Life Sciences, Sun Yat-sen University, Guangzhou, 510006 China; 4 State Key Laboratory of Oncology in South China, Collaborative Innovation Center for Cancer Medicine, Sun Yat-sen University Cancer Center, Guangzhou, 510006 China; 5 State Key Laboratory of Chemical Oncogenomics, School of Chemical Biology and Biotechnology, Peking University Shenzhen Graduate School, Shenzhen, 518055 China

**Keywords:** Phosphorylation, Genomic instability, Cancer therapeutic resistance

## Abstract

Accumulating evidence indicates a correlation between circadian dysfunction and genomic instability. However, whether the circadian machinery directly regulates DNA damage repair, especially in double-strand breaks (DSBs), remains poorly understood. Here, we report that in response to DSBs, BMAL1 is activated by ATM-mediated phosphorylation at S183. Phosphorylated BMAL1 is then localized to DNA damage sites, where it facilitates acetylase CLOCK to load in the chromatin, regulating the acetylation of histone H4 (H4Ac) at DSB sites. In this way, the BMAL1-CLOCK-H4Ac axis promotes the DNA end-resection to generate single-stranded DNA (ssDNA) and the subsequent homologous recombination (HR). BMAL1 deficient cells display defective HR, accumulation of unrepaired DSBs and genome instability. Accordingly, depletion of BMAL1 significantly enhances the sensitivity of adrenocortical carcinoma (ACC) to DNA damage-based therapy in vitro and in vivo. These findings uncover non-canonical function of BMAL1 and CLOCK in HR-mediated DSB repair, which may have an implication in cancer therapeutics.

## Introduction

DNA double-strand breaks (DSBs), which are caused by endogenous agents and exogenous factors including ionizing radiation (IR), are important and potentially cell lethal DNA lesions, which can cause genomic instability, cell-cycle arrest, and even cell death if not repaired in time [1]. There are two major pathways for DSBs repair: homologous recombination (HR)-mediated DSB repair (DSBR) and non-homologous end joining (NHEJ) [2]. HR is a high fidelity DSBR and requires a homologous DNA template compared with NHEJ, which can repair DNA damage in the absence of a homologous DNA template and thus is error prone [3]. DSBR is a complex process that requires strict regulation to coordinate the actions of sensors, transducers and effectors [1, 4]. Importantly, defects in HR-mediated DSBR and NHEJ are related to various human diseases such as immune deficiencies and malignancies [5]. This underscores the significance of DNA damage repair, especially DSBR, for cell survival and human health.

In DSB-induced HR, which occurs after DSB recognition by the MRE11-RAD50-NBS1 (MRN) complex, the CtIP-MRN-BRCA1 complex contributes to DNA end-resection at DSBs to generate the 3′ single-stranded DNA (ssDNA) [6]. Then the ssDNA is bound and protected by RPA protein, which is replaced by RAD51. The ssDNA-RAD51 nucleoprotein filament executes the pairing of homologous DNAs and strand transfer in HR. DNA end-resection plays a crucial role in DSB repair pathway choice, as the ssDNA generated by end-resection is a poor substrate for Ku protein binding and, thus, channels repair towards the HR pathway [7, 8].

Recently, it has been reported that chromatin remodeling and histone modifications are crucial elements in end-resection, suggesting a critical role of histone architecture in guiding DNA damage repair [4, 9]. One of the most important events is the acetylation of the N‐terminal tail of the core Histone H4 [10]. Acetylation of Histone H4 by NuA4 in yeast or TIP60 in mammals is essential for DSB repair and genome stability maintenance [11, 12]. However, whether other acetylases involve in the acetylation of Histone H4 at the DSB sites and elicit DSBR remains poorly understood.

Circadian rhythm affects a lot of physiological processes. Its disruption is implicated in various cellular functions such as DNA damage repair and tumorigenesis through the transcriptional-translational feedback loops (TTFLs) to regulate the oscillation of circadian genes [13–16]. Brain and muscle ARNT-like protein-1 (BMAL1), forming the heterodimer with CLOCK, acts in the positive limb and cryptochrome (CRY1 and CRY2), PER (PER1, PER2, and likely PER3) proteins constitute the repressor arm of the TTFLs to drive the transcription of the circadian-controlled genes. In response to DNA damage, the circadian components have been reported to be involved in Nucleotide Excision Repair (NER) by affecting the oscillation of XPA level [17, 18]. Furthermore, night shift schedule causes circadian dysregulation of DNA repair gene expression, which leads to defects in DNA damage repair and elevates cancer risk [19].

In addition to participating in damage repair by regulating repair factor transcription, emerging evidences show its direct engagement in DNA damage repair. For example, CRY1/TIM interact with Chk1 to regulate the activation of ATR-Chk1 [20]. PER1 interacts with the ATM/Chk2 complex to regulate DNA damage repair in response to DSBs [21]. These reports show that the negative elements of circadian rhythm can directly participate in the DNA damage repair. However, whether positive elements of circadian rhythm, such as BMAL1 and CLOCK, are directly engaged in DNA damage repair, especially DSBR, has not been addressed yet. In this study, we uncovered a non-canonical function of BMAL1 in DSBs repair and tumor chemoresistance. We found that BMAL1 is phosphorylated by ATM in response to DSBs. Evidence is presented that S183-p-BMAL1 is recruited to DSBs leading to the recruitment of CLOCK, which acetylates the Histone H4 to promote the DNA end-resection and HR. Importantly, clinical studies indicate that high expression of BMAL1 correlates with poor prognosis for cancer patients. High expression of BMAL1 in cancer cells could decrease sensitivity/increase resistance to oncotherapeutic agents and result in poor cancer treatment outcomes.

## Results

### ATM mediates phosphorylation of BMAL1 serine 183 and localization to DSB sites

**Fig. 1:**
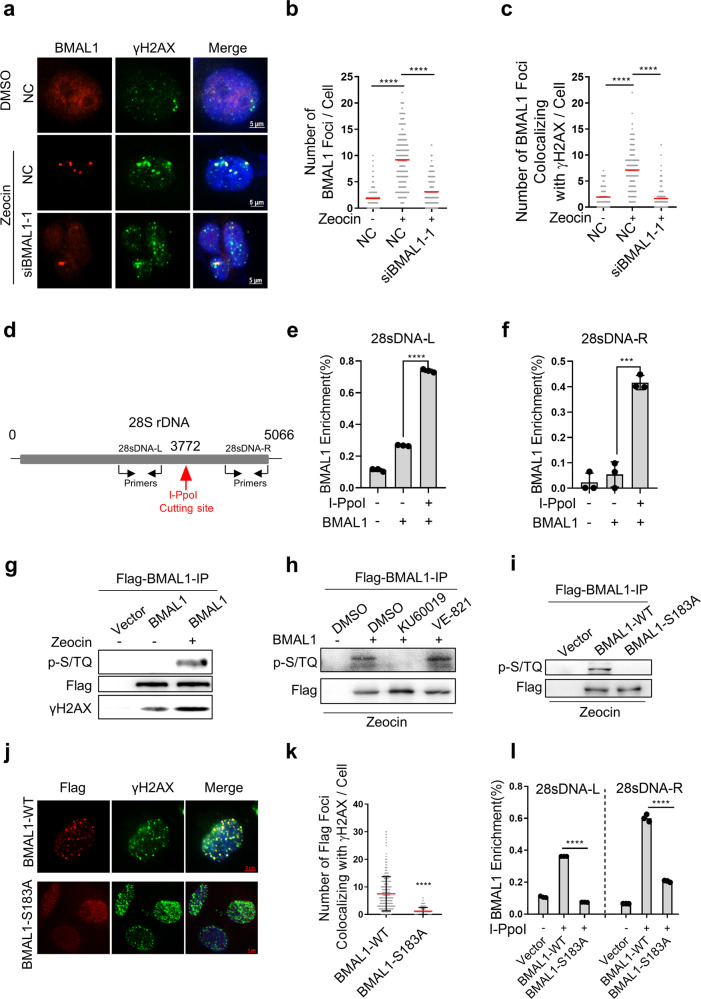
ATM-dependent phosphorylation of BMAL1. **a** Immunofluorescence detection of BMAL1 and γH2AX foci in U2OS cells transfected with NC or siBMAL1. Cells were treated with Zeocin or DMSO. Scale bar: 5 μm. **b** Quantification of panel **a**. The number of BMAL1 foci per cell (*n* ≥ 100). **c** Quantification of panel **a**. The number of BMAL1 foci colocalized with γH2AX foci. **d** Location of I-PpoI cutting site at 28S rDNA. The positions of primers used for ChIP-qPCR are indicated by arrows. **e**, **f** ChIP-qPCR for 28S rDNA with or without cutting by I-PpoI and binding by BMAL1. **g** Flag-BMAL1-overexpressing HEK293T cells were treated with Zeocin or DMSO, followed by pulldown with anti-Flag beads and immunoblotting with antibodies against p-S/TQ, Flag and γH2AX. **h** Flag-BMAL1-overexpressing HEK293T cells with or without ATM inhibitor KU60019 or ATR inhibitor VE-821 pretreatment were treated with Zeocin, followed by pulldown with anti-Flag beads and immunoblotting with antibodies against p-S/TQ and Flag. **i** HEK293T cells with Zeocin pretreatment were overexpressed Flag-Wild type BMAL1 (BMAL1-WT) or Flag-S183A BMAL1 (BMAL1-S183A), followed by pulldown with anti-Flag beads and immunoblotting with antibodies against p-S/TQ and Flag. **j** Immunofluorescence detection of Flag and γH2AX foci in U2OS cells overexpressed with Flag-BMAL1-WT or Flag-BMAL1-S183A. Cells were treated with Zeocin. Scale bar: 5 μm. **k** Quantification of panel **j**. The number of Flag foci colocalized with γH2AX foci. **l** ChIP-qPCR for 28S rDNA with or without cutting by I-PpoI and binding by BMAL1-WT or BMAL1-S183A. All values are the average ± SEM of three independent experiments. Student’s unpaired two-tailed t-test was used to determine the statistical significance (^***^P < 0.001; ^****^P < 0.0001).

To find out whether BMAL1 directly participates in DSBR, human sarcoma U2OS cells were treated with Zeocin, a radio-mimetic chemical that predominantly induces DSBs [22, 23], and the localization of BMAL1 was examined by immunofluorescence (IF). Strikingly, compared with undamaged cells, BMAL1 formed distinct nuclear foci after exposure to Zeocin colocalized with γH2AX foci, the marker of DSBs [24, 25]. In addition, the BMAL1 foci were decreased after knockdown of BMAL1 by siRNA, indicating that the signals from the BMAL1 antibody are specific (Fig. 1a–c, Supplementary Fig. 1a). This idea was further confirmed by engineering HEK293T cells to carry a specific target cleavage site in 28S rDNA and DAB1 for I-PpoI [26]. After generating a site-specific DSB in 28S rDNA and DAB1 in these cells, the binding efficiency of BMAL1 to DSB sites was examined by ChIP followed by q-PCR (Fig. 1d, Supplementary Fig. 1b). We found I-PpoI-dependent induction of DSBs at 28S rDNA and DAB1 leading to decrease of PCR product (uncut DNA) and more BMAL1 binding (Fig. 1e, f, Supplementary Fig. 1c, d). In addition, we used several DSB-induced drugs, which mainly activate the ATM (ataxia-telangiectasia mutated) kinase [27, 28], or several single-strand break (SSB)-induced agents, which mainly activate the ATR (Ataxia Telangiectasia and Rad-3-related protein) kinase [29, 30], to treat the cells and examined the expression level of BMAL1 (Supplementary Fig. 1e). BMAL1 was significantly up-regulated after DSB-induced drugs treatment compared with SSBs-induced agents (Supplementary Fig. 1e). And the expression level of BMAL1 gradually increased with the increased treatment time or concentration of Zeocin (Supplementary Fig. 1f, g). These results suggested that BMAL1 is specially involved in highly-coordinated DDR in response to DSBs.

The ATM phosphorylates various substrates to maintain genome integrity during DNA damage. Given our results identifying BMAL1 could be recruited to DSB sites and be up regulated by DSB drugs, we further investigated whether BMAL1 would be phosphorylated by ATM kinase upon DSBs. As shown in (Fig. 1g), following Zeocin treatment, BMAL1 was phosphorylated at its SQ/TQ motifs, which are consensus ATM/ATR phosphorylation sites [31]. In addition, the BMAL1 could interact more with γH2AX upon Zeocin treatment, which is consistent with the result of BMAL1 localized to DSB sites. This phosphorylation is dependent on ATM, as treatment with the pharmacological ATM inhibitor (KU60019) but not the ATR inhibitor (VE-821) reduced this signal (Fig. 1h). To confirm whether this phosphorylation is DSBs specific, we treated the BMAL1 overexpressed cells with DSB-induced drugs (Zeocin, VP-16) or SSB-induced agents (CPT, H_2_O_2_, UVC) and found that only DSB drugs could promote the phosphorylation of BMAL1 (Supplementary Fig. 1h). In silico analysis revealed that serine 183 of BMAL1 at its PAS domain is a conserved ATM/ATR phosphorylation consensus motif ((S/T)Q), which is highly conserved in vertebrates (Supplementary Fig. 1i). To confirm whether S183 is a phosphorylation site, we constructed the phosphodeficient mutant (S183A) of BMAL1 and found that the S183A of BMAL1 abolished the p(S/T)Q signal (Fig. 1i).

Furthermore, ATM-mediated BMAL1 phosphorylation appears to play a specific role in DDR, because BMAL1 failed to localize to DSB sites upon pre-treated the cells with KU60019 but not the VE-821 (Supplementary Fig. 1j–l). In a similar manner, BMAL1 with an S183A point mutation failed to localize to DSB sites in response to DNA damage (Fig. 1j–l, Supplementary Fig. 1m, n), suggesting that ATM-dependent phosphorylation of BMAL1 S183 is required for localization of BMAL1 to DSBs.

### BMAL1 promotes HR-mediated DSBR

**Fig. 2:**
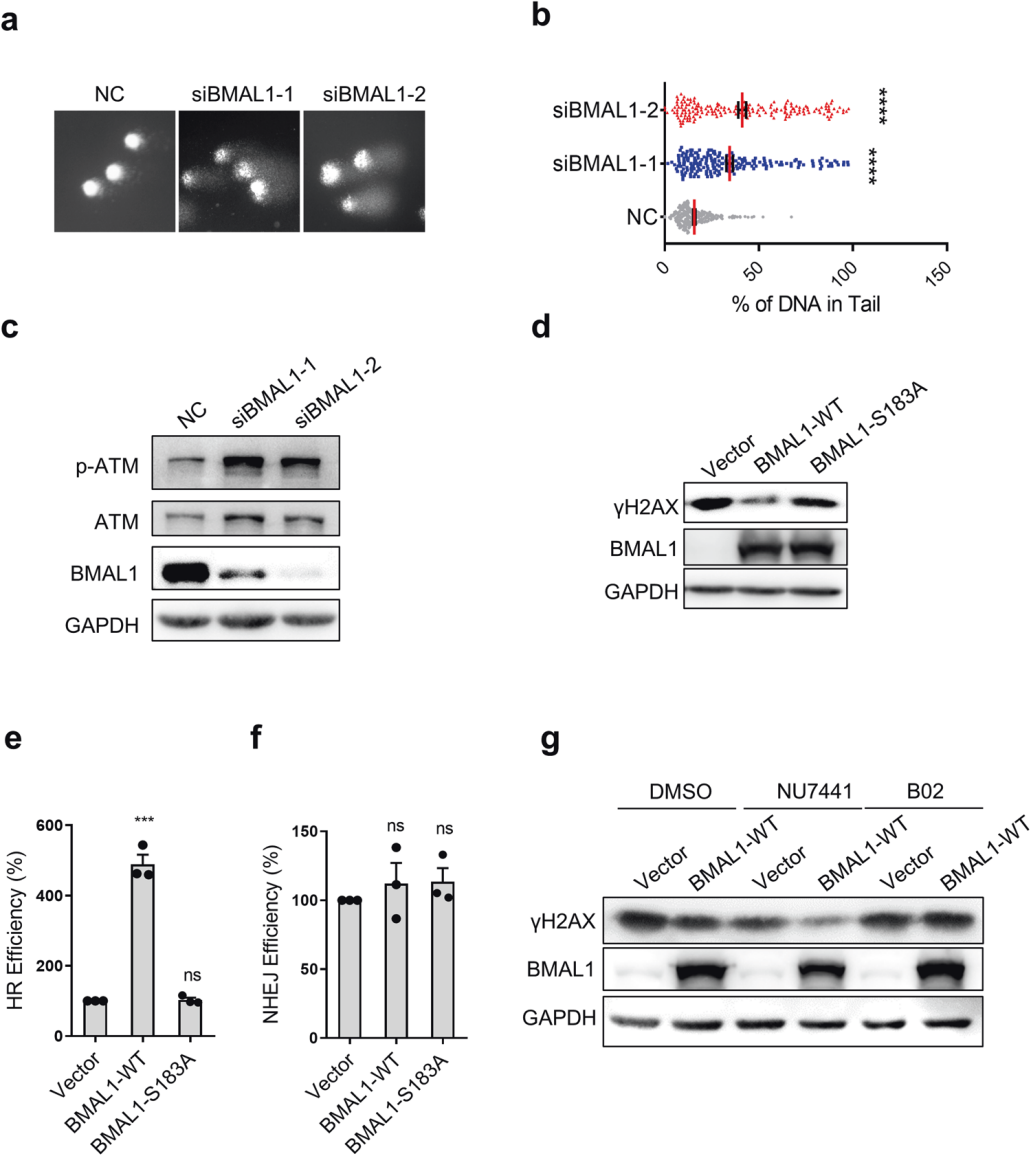
BMAL1 is crucial for HR-mediated DSBR. **a** Representative results of neutral comet assay. U2OS cells were transfected with siNC, siBMAL1-1 or siBMAL1-2. **b** Quantification of panel **a**. The percentages of DNA in the tail (*n* ≥ 100). **c** Immunoblot analysis of phosphorylated ATM (p-ATM), total ATM and BMAL1 in U2OS cells transferred with siNC, siBMAL1-1 or siBMAL1-2. **d** Immunoblot analysis of γH2AX and BMAL1. HEK293T cells over-expressed with BMAL1-WT or BMAL1-S183A were treated with Zeocin and incubated in fresh culturing medium for 6 h. **e** FACS analysis the frequency of HR in BMAL1-WT or BMAL1-S183A overexpressed U2OS DR‐GFP cell line transfected with I-SceI. **f** FACS analysis the frequency of NHEJ in BMAL1-WT or BMAL1-S183A overexpressed U2OS EJ5-GFP cell line transfected with I-SceI. **g** Immunoblot analysis of γH2AX and BMAL1. B02 or NU7441 or DMSO was added to culturing medium and cells over-expressing vector or BMAL1-WT were collected 6 h after Zeocin treatment. All values are the average ± SEM of three independent experiments. Student’s unpaired two-tailed t-test was used to determine the statistical significance (^***^P < 0.001, ^****^P < 0.0001).

We then explored how depletion of BMAL1 affects DSBR. BMAL1 was knocked down in U2OS, the amount of DSBs was determined by detecting DNA fragments released by DSBs and the level of phosphorylation of ATM. Depletion of BMAL1 significantly increased the broken DNA fragments as detected by comet assay and constant-field gel electrophoresis (CFGE) and leaded to persistence of phosphorylation of ATM [32] (Fig. 2a–c, Supplementary Fig. 2a, b). Consistently, overexpression of BMAL1-WT, but not BMAL1-S183A accelerated DSBR (Fig. 2d). Whereas, lack of BMAL1 in cells slowed down the repair of DNA damage (Supplementary Fig. 2c). These results suggest that BMAL1 localizing to DSBs is required for DSBR.

DSBs are mainly repaired by error-free HR or error-prone NHEJ. To investigate whether BMAL1 promotes one or the other of these pathways (or both), we used the DR-GFP or EJ5-GFP system to examine the HR or NHEJ efficiency, resepectly [33, 34]. We first overexpressed BMAL1-WT or BMAL1-S183A in DR-GRP or EJ5-GFP U2OS cells and measured the efficiency of DSB repair by HR or NHEJ through FACS. The results showed that overexpression of BMAL1-WT but not the BMAL1-S183A significantly promotes HR-mediated DSBR while having no effect on NHEJ-mediated DSBR (Fig. 2e, f). Moreover, knockdown of BMAL1 impaired HR-mediated DSBR, while having no effect on NHEJ-mediated DSBR (Supplementary Fig. 2d, e). To further confirm the role of BMAL1 in HR, we used the B02 [35] or NU7441 [36] to specifically inhibit HR or NHEJ, respectively. As shown in (Fig. 2g), over-expression of BMAL1 failed to stimulate the repair of Zeocin-induced DSBs in the presence of B02. Meanwhile, NU7441 has no effect on BMAL1-stimulated repair of Zeocin-induced DSBs. To this end, to exclude the possibility that p-BMAL1 enhances the DBSR through promoting the transcription of DNA damage repair factor, we texted the expression level of gene downstream of BMAL1 by RT-qPCR. The result showed that the S183A mutation does not affect the transcriptional activity of BMAL1 (Supplementary Fig. 2f).

### BMAL1 stimulates DNA end-resection

**Fig. 3:**
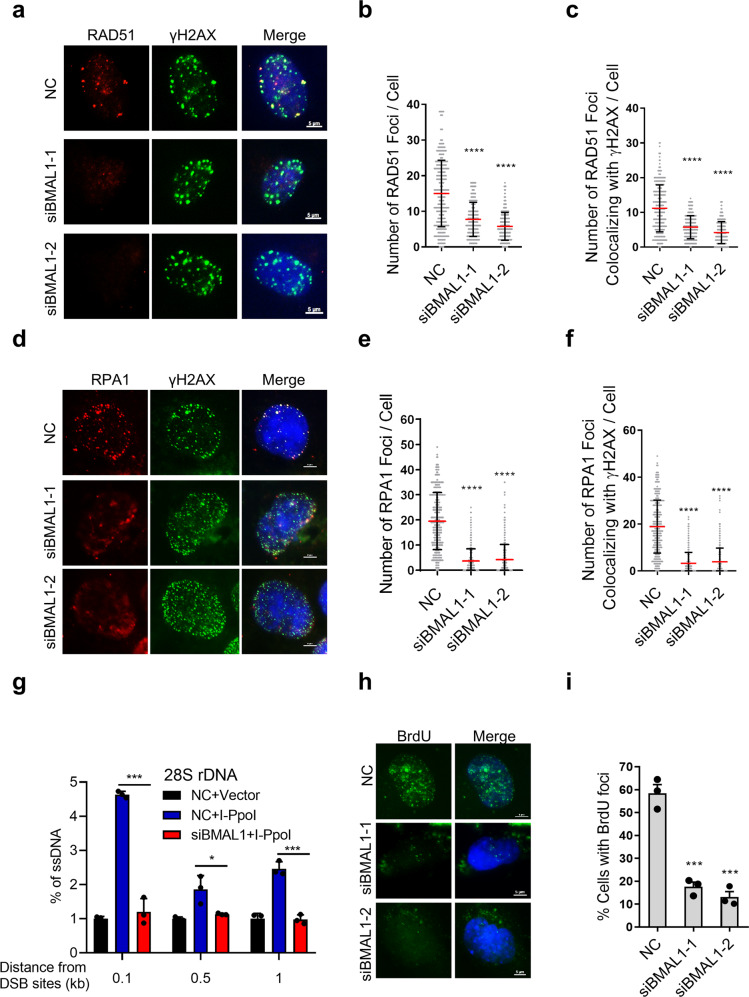
Loss of BMAL1 impairs the recruitment of HR repair factor and DNA end resection. **a** Immunofluorescence detection of RAD51 and γH2AX foci in U2OS cells transfected with NC, siBMAL1-1 or siBMAL1-2. Cells were treated with Zeocin. Scale bar: 5 μm. **b** Quantification of panel **a**. The number of RAD51 foci per cell (*n* ≥ 100). **c** Quantification of panel **a**. The number of RAD51 foci colocalized with γH2AX foci. **d** Immunofluorescence detection of RPA1 and γH2AX foci in U2OS cells transfected with NC, siBMAL1-1 or siBMAL1-2. Cells were treated with Zeocin. Scale bar: 5 μm. **e** Quantification of panel **d**. The number of RPA1 foci per cell (*n* ≥ 100). **f** Quantification of panel **d**. The number of RPA1 foci colocalized with γH2AX foci. **g** Detection of the abundance of ssDNA in BMAL1 depleted HEK293T cells transfected with I-PpoI. qPCR was performed on the genomic DNA with the indicated primers amplifying regions including the positions at 0.1, 0.5 and 1 kb downstream from the DSB, as depicted. The values of %ssDNA was calculated as follows: %ssDNA=1/[(2ΔCt-1)+0.5] × 100. **h** Immunofluorescence detection of BrdU foci in U2OS cells transfected with NC, siBMAL1-1 or siBMAL1-2. Cells were treated with Zeocin. Scale bar: 5 μm. **i** Quantification of panel **h**. The percentage of cell with BrdU foci (*n* ≥ 100). All values are the average ± SEM of three independent experiments. Student’s unpaired two-tailed t-test was used to determine the statistical significance (^*^P < 0.05, ^***^P <0.001; ^****^P < 0.0001).

DNA end-resection is the key event that controls the DSB repair pathway choice, which generates the long ssDNA leading cells prone to HR-mediated repair [37]. Because BMAL1 promotes HR and has no effect on NHEJ, we speculated that BMAL1 might regulate the DNA end-resection. To test this, we performed IF to examine the localization of RAD51 and RPA1 at DSB sites, which are the key factors of HR and indicators of the amount of ssDNA [38]. The results showed that the RAD51 and RPA1 foci are decreased upon knockdown of BMAL1 in U2OS cells treated with Zeocin (Fig. 3a–f). In addition, overexpression of BMAL1-WT but not S183A mutation increases the RAD51 and RPA1 foci at DSB sites (Supplementary Fig. 3a–f). Importantly, we also demonstrated that both knockdown and overexpression of BMAL1 do not affect expression of RAD51 and RPA1 (Supplementary Fig. 3g, h). Failure to generate RAD51 and RPA1 foci by knockdown of BMAL1 could be associated with either a defect in end-resection of the DSB into ssDNA, or with impaired recruitment of RAD51 and RPA1 itself to ssDNA. To distinguish between these two possibilities, we tested the efficiency of ssDNA generation in BMAL1-depleted cells exposed to DNA damage. We used I-PpoI to generate a site-specific DSB in 28S rDNA, and then extracted genomic DNA for enzyme digestion by duplex-specific nuclease (DSN), a nuclease specific for double-stranded DNA [39], the level of ssDNA at 28S rDNA was examined by qPCR (Supplementary Fig. 3i). Consistent with the previous observation, I-PpoI-dependent induction of DSBs at 28S rDNA leads to increase of ssDNA [40, 41], which demonstrated that the system used in this study can faithfully represent the level of ssDNA. Furthermore, we found that the level of ssDNA induced by DSB was decreased when the BMAL1 was knocked down (Fig. 3g, Supplementary Fig. 3j). To further confirm the role of BMAL1 in DNA end-resection, we labeled the U2OS cells with BrdU and subsequently performed IF staining the BrdU under non-denaturing conditions to monitor ssDNA levels. As shown in (Fig. 3h, i), the percentage of cells with BrdU foci was significantly reduced in BMAL1-deficient cells. In addition, overexpression BMAL1-WT but not the S183A mutant increased the level of ssDNA (Supplementary Fig. 3k, l), indicating that ATM-mediated phosphorylation of BMAL1 is required for the efficient generation of RPA-coated ssDNA at resected DSBs. Altogether, these results suggest that BMAL1 directly promotes HR through regulating the process of DNA end-resection.

### CLOCK is recruited to DSB sites by BMAL1 and promotes histone acetylation

**Fig. 4:**
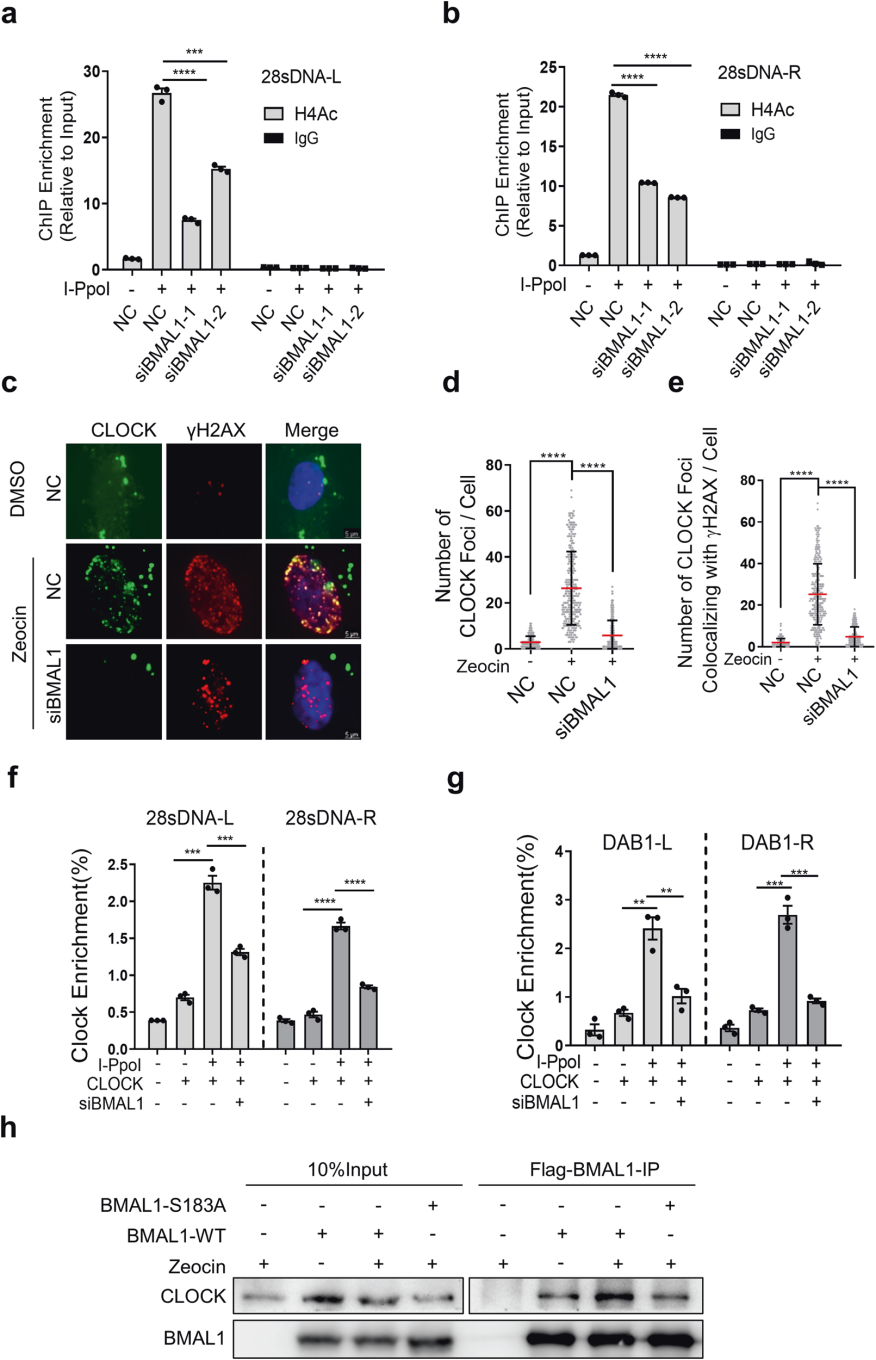
BMAL1 regulates Histone H4 acetylation and the recruitment of CLOCK to DSBs. **a**, **b** ChIP-qPCR showing the relative enrichment of Histone H4 acetylation (H4Ac) at 28S rDNA with or without cutting by I-PpoI. HEK293T cells were transfected with siNC, siBMAL1-1 or siBMAL1-2. **c** Immunofluorescence detection of CLOCK and γH2AX foci in U2OS cells transfected with NC or siBMAL1. Cells were treated with Zeocin or DMSO. Scale bar: 5 μm. **d** Quantification of panel **c**. The number of CLOCK foci per cell (*n* ≥ 100). **e** Quantification of panel **c**. The number of CLOCK foci colocalized with γH2AX foci. **f** ChIP-qPCR for 28S rDNA with or without cutting by I-PpoI and binding by CLOCK. HEK293T cells were transfected with siNC or siBMAL1. **g** ChIP-qPCR for DAB1 with or without cutting by I-PpoI and binding by CLOCK. HEK293T cells were transfected with siNC or siBMAL1. **h** Co-IP assay to determine the interaction of Flag-BMAL1-WT or Flag-BMAL1-S183A with CLOCK in control and Zeocin treated HEK293T cells. HEK293T cells were immunoprecipitated with Flag-beads. All values are the average ± SEM of three independent experiments. Student’s unpaired two-tailed *t*-test was used to determine the statistical significance (^**^P < 0.01, ^***^P <0.001; ^****^P < 0.0001).

We then explored how depletion of BMAL1 affects end-resection. As chromatin remodeling regulated by histone modification, in particular acetylation of Histone H4 (H4Ac), is critical for DNA end-resection in eukaryotes and HR-mediated DSBs repair [12, 42, 43]. CLOCK has an intrinsic acetyltransferase domain that enables chromatin remodeling by acetylating Histones H4 to promote the transcription of circadian CLOCK-controlled genes [44, 45]. In addition, Cotta-Ramusino et al. reported that CLOCK also locates to DNA damage sites, but the function and localization mechanism of CLOCK at DNA damage sites are still unknown [46]. Accordingly, we speculated that CLOCK might be recruited by BMAL1 to DSB sites, leading the Histone H4 acetylation modification and promoting end-resection. Thus, we first determined whether loss of BMAL1 affects the level of H4Ac at DSB sites. By ChIP using H4Ac antibody coupled with qPCR, we found that H4Ac is enriched at 28 s rDNA and DAB1 sites in response to DSBs induced by I-PpoI [42]. Moreover, after depletion of BMAL1, the level of H4Ac decreased significantly (Fig. 4a, b, Supplementary Fig. 4a–c). To find out whether CLOCK collaborates with BMAL1 to promote DSBR, we performed IF to examine the localization of CLOCK upon Zeocin treatment. As expected, our results demonstrated the CLOCK can be recruited to DSB sites, in addition, we also found that this recruitment is BMAL1-dependent (Fig. 4c–g, Supplementary Fig. 4d). Furthermore, we performed co-IP experiment to examine the interaction between BMAL1 and CLOCK in the context of DSBs. It was found that in response to Zeocin treatment, CLOCK is more precipitated by BMAL1-WT, but not BMAL1-S183A (Fig. 4h). However, the localization of BMAL1 at DSB sites was not affected by depletion of CLOCK (Supplementary Fig. 4e–i). These results suggest that the BMAL1 may promote DSBR through recruiting CLOCK to the damage sites.

**Fig. 5:**
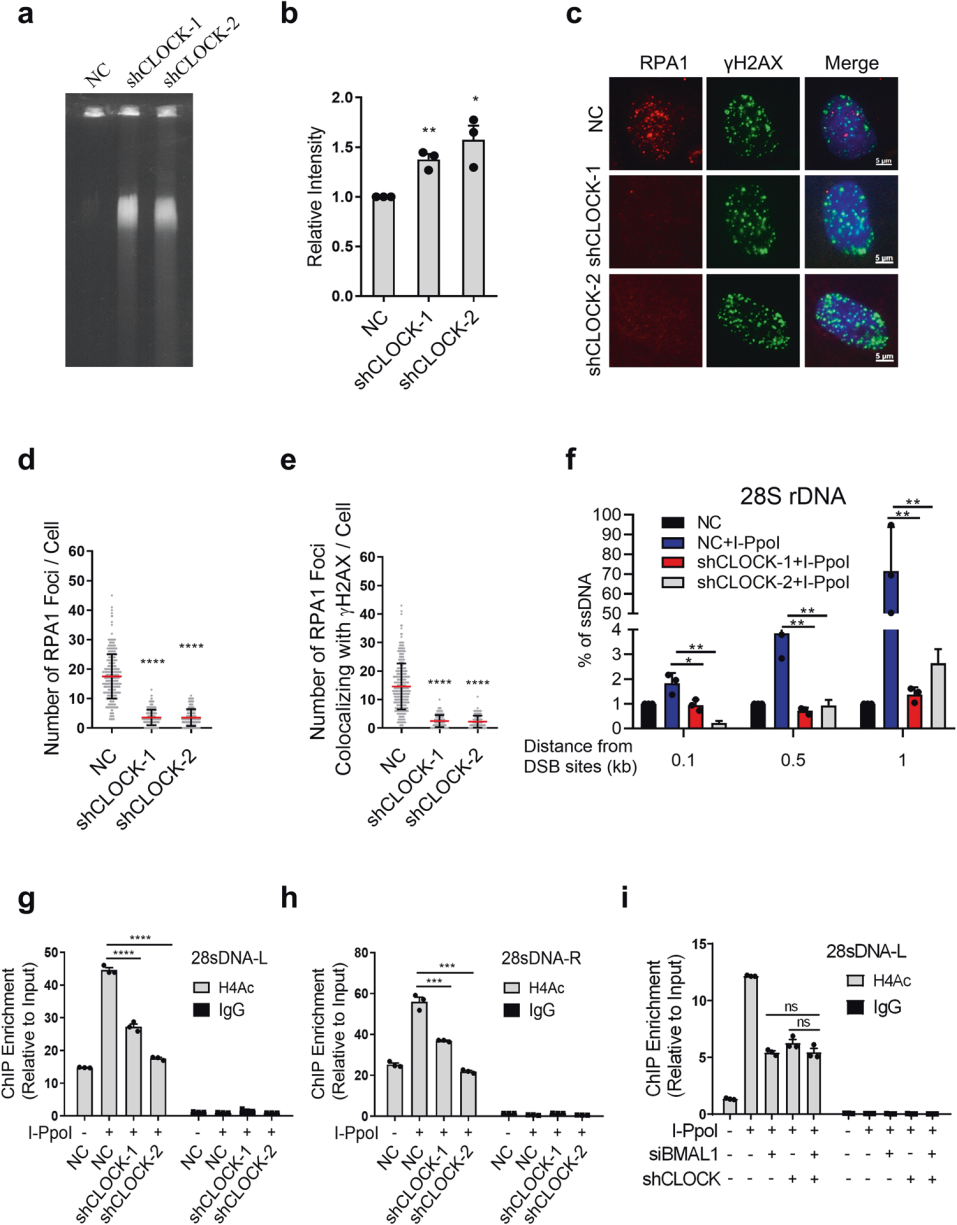
Loss of CLOCK impairs the H4Ac and DNA end-resection at DSBs. **a** Constant-field gel electrophoresis (CFGE) analysis of DNA fragment released by DSBs on genome in normal and CLOCK depleted U2OS (shCLOCK-1, shCLOCK-2). Gel was stained with GelRed. **b** Quantification of **a**. The relative amount of DNA fragments was determined by normalizing to the smear signal of cells transfected with NC. **c** Immunofluorescence detection of RPA1 and γH2AX foci in normal and CLOCK depleted U2OS (shCLOCK-1, shCLOCK-2). Cells were treated with Zeocin. Scale bar: 5 μm. **d** Quantification of panel **c**. The number of RPA1 foci per cell (*n* ≥ 100). **e** Quantification of panel **c**. The number of RPA1 foci colocalized with γH2AX foci. **f** Detection of the abundance of ssDNA in CLOCK depleted HEK293T cells (shCLOCK-1, shCLOCK-2) transfected with I-PpoI. qPCR was performed on the genomic DNA with the indicated primers amplifying regions including the positions at 0.1, 0.5 and 1 kb downstream from the DSB, as depicted. The values of %ssDNA was calculated as follows: %ssDNA=1/[(2ΔCt-1)+0.5] × 100. **g**, **h** ChIP-qPCR showing the relative enrichment of Histone H4 acetylation (H4Ac) at 28S rDNA with or without cutting by I-PpoI. HEK293T cells were transfected with NC, shCLOCK-1 or shCLOCK-2. **i** ChIP-qPCR showing the relative enrichment of Histone H4 acetylation (H4Ac) at 28S rDNA with or without cutting by I-PpoI. HEK293T cells were transfected with indicated siRNAs or shRNAs. All values are the average±SEM of three independent experiments. Student’s unpaired two-tailed t-test was used to determine the statistical significance (^*^P < 0.05, ^**^P < 0.01, ^***^P < 0.001, ^****^P < 0.0001).

We thus explored whether CLOCK is involved in DSBR. The CFGE and immunoblot assay on control and CLOCK deficient cells demonstrated that CLOCK promotes the DSBR, because depletion of CLOCK leads to accumulation of DNA fragments and increased amount of p-ATM indicating unrepaired DSBs (Fig. 5a, b, Supplementary Fig. 5a). Furthermore, the RPA1 foci and the level of ssDNA at DSB sites are decreased upon knockdown of CLOCK (Fig. 5c–f, Supplementary Fig. 5b, c), which further proves that CLOCK participates in DSBR. Then we verified whether CLOCK is involved in the regulation of H4Ac modification at DSB sites. Through H4Ac-ChIP, we found that depletion of CLOCK inhibits the acetylation of Histone H4 at DSBs (Fig. 5g, h, Supplementary Fig. 5d, e). In addition, knockdown of BMAL1 and CLOCK at the same time did not further promote the H4Ac modification decreased caused by BMAL1 or CLOCK absence (Fig. 5i, Supplementary Fig. 5f), which suggests that the H4Ac modification at DSBs mediated by CLOCK is at the same pathway with BMAL1. To determine whether the acetyltransferase activity of CLOCK is required for its regulatory role in end-resection and HR, we further generated CLOCK mutants, which have been reported to exhibit reduced acetyltransferase activity due to the substitution of three amino acids [44, 47]. Subsequent IF analysis revealed that introduction of exogenous wild-type CLOCK (CLOCK-WT) but not mutational CLOCK (CLOCK-MUT) into CLOCK depleted U2OS effectively rescues the ability of HR, as evidenced by the increased RPA1 foci (Supplementary Fig. 5g–j). These results suggest that CLOCK antagonizes DNA damage depending on its acetyltransferase activity.

### BMAL1-deficiency increases the sensitivity of tumors to DNA damage reagents

**Fig. 6:**
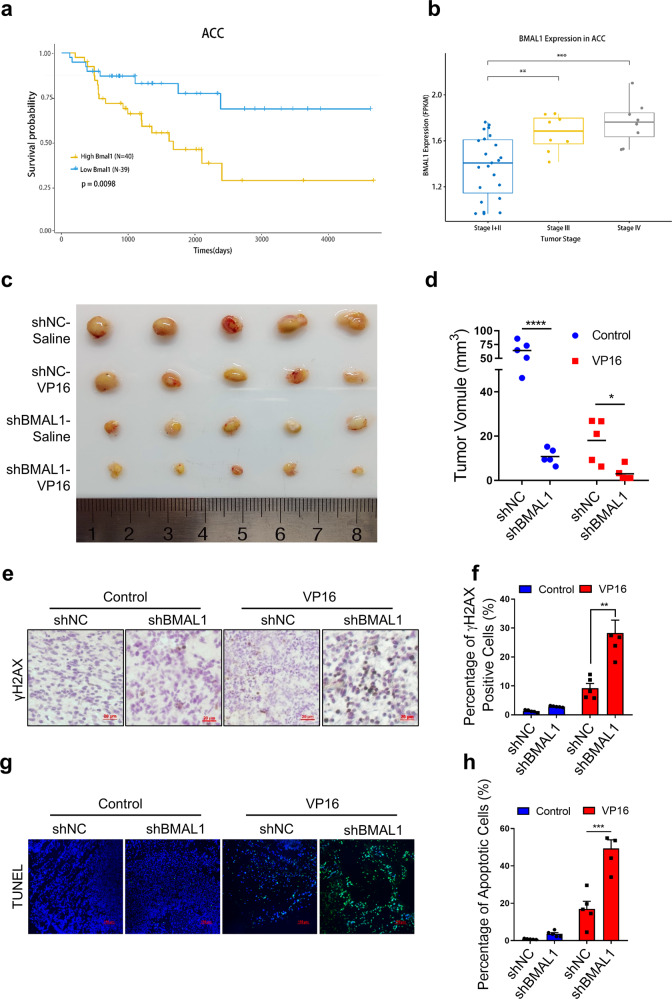
BMAL1 deficiency sensitizes tumors to DNA damage reagents. **a** Kaplan–Meier curve analysis of the overall survival probabilities of ACC patients. Patients were assigned to two groups based on low or high levels of BMAL1 expression. **b** The relative expression level of BMAL1 in specimens from patients with stage I to IV ACC. Data were obtained from the TCGA database. **c** Mice were injected subcutaneously with 1 × 10^7^ normal (shNC) or BMAL1 stably depleted (shBMAL1) SW-13 cells and treated with 5 mg/kg of VP16 or vehicle control every two days starting on day 0 for 8 days. Representative images of xenograft tumors were showed. **d** Quantification of **c**. The volume of xenograft tumors (*n* = 5 per group). **e** Representative histology staining images showing the signal of γH2AX in VP16 treated and untreated xenograft tumors driven by normal or BMAL1 depleted SW-13 cells. Scale bar: 20 μm. **f** Quantification of **e** to determine the percentage of γH2AX positive cells. **g** Representative histology staining images showing the apoptotic cells in VP16 treated and untreated xenograft tumors driven by normal or BMAL1 depleted SW-13 cells. Scale bar: 100 μm. **h** Quantification of **g** to determine the percentage of apoptotic cells. For each group, >1000 cells were examined. All values are the average±SEM of three independent experiments. The unpaired Student’s two-tailed *t*-test was used to determine the statistical significance (^*^P < 0.05, ^**^P < 0.01, ^***^P < 0.001, ^****^P < 0.0001).

It is well recognized that cancer cells that are deficient in HR-mediated DSBR are frequently more sensitive to killing by chemotherapy drugs such as etoposide or irradiation than the corresponding cells that are proficient in HR-DSBR [48, 49]. Therefore, inhibition of DDR protein activity and the combination of radiation/chemo-therapeutic agents can help to kill highly proliferating cancer cells. We observed that knockdown of BMAL1 significantly increases the level of micronuclei upon Zeocin treatment (Supplementary Fig. 6a, b), which suggests induction of genome instability. Furthermore, SW-13 cell, the adrenocortical carcinoma (ACC), depleted BMAL1 increases the sensitivity to Zeocin, and that overexpression of BMAL1-WT, but not BMAL1-S183A, increases the resistance of SW-13 cell to Zeocin (Supplementary Fig. 6c–f). Clinical data also suggests that expression level of BMAL1 is up-regulated with the increase of ACC malignancy and that higher expression of BMAL1 predicts poor survival probability for patients (Fig. 6a, b). Thus, it is tempting to speculate that BMAL1 may have prognostic value with respect to therapeutic outcome for patients with ACC.

We then determined the effect of BMAL1 loss on ACC growth and chemotherapy tolerance in vivo by a murine xenograft model. The results showed that depletion of BMAL1 significantly inhibits tumor growth in control animals injected with vehicle, as expected, and tumor growth is even more strongly suppressed in VP16-treated recipient mice carrying BMAL1-depleted xenograft tumors (Fig. 6c, d). The body weight of xenograft tumor-bearing mice remained stable in all treatment groups (Supplementary Fig. 6g). The abundance of γH2AX was slightly higher in BMAL1-deficient xenograft tumors, and they increased to much higher level after VP16 treatment and increased rate of tumor cell apoptosis (Fig. 6e–h). These results indicate that depletion of BMAL1 strongly sensitizes the xenograft tumor to VP16-induced DNA damage, leading to massive tumor cell death and improving therapeutic outcome in mouse xenograft model system.

## Discussion

DSBs trigger a genome reconnaissance pathway coordinated essentially by ATM, an expert controller of DDR whose downstream effectors advance DNA repair and deferral or capture cell-cycle movement [27, 28]. Whereas, DSBR is a complex process involving many signaling pathways and is not completely known. Here, we uncovered a direct participation role of BMAL1-CLOCK-H4Ac in DSBR. We observed that BMAL1 is phosphorylated/activated by ATM at S183 in response to DSBs. The activated BMAL1 localizes to DNA damage sites and promotes HR-mediated DSBR independent of its transcriptional activity. Mechanistically, BMAL1 regulates the localization of CLOCK to DSB sites to acetylize the Histone H4 and promotes the end-resection to generate the ssDNA, which then recruits RAP1 and RAD51 for HR.

### Activation of BMAL1 by phosphorylation at S183

It is reported that BMAL1 undergoes post-translational modifications such as phosphorylation, which plays a key role in regulating the dynamics of circadian rhythms. Interestingly, phosphorylation of these serine/threonine residues in BMAL1 regulates the transcription activity through modulation of protein turnover [50–52]. Our study shows that ATM-dependent DNA damage-induced phosphorylation of BMAL1 at S183 is required for BMAL1 to stimulate DSBR. Because mutation of BMAL1 S183 to A has no effect on the transcription activity of BMAL1 but impairs its localization to DSB sites (Fig. 1a, j–l, Supplementary Fig. 1m, Supplementary Fig. 2f and Supplementary Fig. 3g, h). Further investigation is needed to elucidate this process in molecular detail, including the mechanism that BMAL1 with phosphorylation sites mutation fails to localize to DSB sites.

### Direct role of BMAL1 in DSBR

In the past few decades, the circadian proteins such as BMAL1 have been heavily studied as circadian transcriptional regulators and regulate gene expression involved in DDR [18, 19, 53]. In the DSBs repair, Palombo et al. found higher DNA repair activities for IR-induced DNA damage in mouse splenocytes during the light phase (ZT06) than those of the dark phase (ZT18). Gene profiling reveals that the majority of the DNA repair genes were expressed at higher levels during the light phase including BRCA1 and BRCA2, two genes involved in HR, suggesting the circadian protein control of HR at transcriptional level [54].

Except affecting DDR through transcriptional regulation, it is also found that the key circadian factors may play a specific role in DDR by participating as a key modulator. For instance, CRY1/TIM form a protein complex with Chk1 and this interaction is required for ATR-Chk1 kinase activation [20]. PER1 interacts with the ATM/Chk2 complex to regulate DNA damage repair in response to DSBs [21]. In addition, recent finding uncovered BMAL1 forms complexes with Lamin B1, KAP1 and HP1α to stabilize heterochromatin and protect mesenchymal progenitor cells against senescence [55]. All of these suggest that circadian factors have a non-canonical role in DNA damage repair or genomic stability maintenance.

Here, we discovered that BMAL1 plays direct role in promoting HR-mediated DSBR, as suggested by the following observations. First, ATM-mediated phosphorylation of BMAL1 at S183 is specifically activated by DSBs (Fig. 1g–i) and S183 phosphorylated BMAL1 is recruited to DSBs, implying its direct involvement in DSBR (Fig. 1j–l, Supplementary Fig. 1j–m). Second, overexpression of phosphorylation defect BMAL1, which has a normal transcriptional activity in downstream factors (Supplementary Fig. 2f), is unable to increase the efficiency of DDR (Fig. 2d, e), suggesting that canonical functions of BMAL1 on transcription have a minor effect on DSBR. Third, BMAL1-CLOCK complex promotes the DNA end-resection and recruitment of HR proteins to DSBs (Fig. 3, Fig. 4 and Fig. 5). Fourth, depletion of BMAL1 does not change the expression level of RAD51 and RPA1, thus excluding the possibility that BMAL1 promotes HR via upregulating the expression of RAD51 or RPA1 (Supplementary Fig. 3g, h).

### H4Ac in HR-mediated DSBR

Chromatin creates a natural barrier against access to DNA during transcription, damage repair and recombination. There is an increasing body of evidence about the role of histone acetylation in DSB repair and maintenance of genome stability, but the Histone H4 acetylase involved in DSBR remains partially understood. And the CLOCK, a Histone H4 acetylase, has been reported to be able to colocalize with γH2AX [46] and stabilize heterochromatin [47], which suggests that it may play a direct role in the DDR. However, the function of CLOCK in DDR and the mechanism of CLOCK being recruited to the sites of DNA damage have not been thoroughly studied. Here, we reveal increased interaction of CLOCK with BMAL1 after Zeocin treatment and specific recruitment to DSB sites depending on BMAL1, where it acetylates Histone H4 and ensures the process of end-resection. Furthermore, the histone acetyltransferase activity-deficient CLOCK mutant decreases the process of end-resection to generate ssDNA (Fig. 5).

### Role of BMAL1 in cancer therapeutics

Increased evidence indicates the association between the BMAL1 dysfunction and the development and proliferation of tumors. In our finding, the clinical studies show that expression level of BMAL1 is up-regulated with the increase of ACC malignancy and higher expression of BMAL1 correlates with lower survival probability in ACC patients. (Fig. 6a, b). Consistent with these observations, in vitro studies show that depletion of BMAL1 significantly increases the sensitivity of ACC cancer cells to Zeocin treatment (Supplementary Fig. 6c). In addition, depletion of BMAL1 strongly suppresses xenograft tumor growth in mice treated with VP16 (Fig. 6c–h). These results are consistent with the previous findings that depletion of BMAL1 sensitized breast cancer cells to cisplatin and doxorubicin [56] and suggest that BMAL1 may have potential as a therapeutic target, because its inhibition or depletion could improve cancer treatment outcomes. However, Zeng et al. reported that down-regulation of BMAL1 accelerates the growth of murine colon cancer in vivo and in vitro and decreased its apoptosis induced by VP-16 [57]. This discrepancy is common since cancer is a complex and heterogeneous genomic disease with distinct molecular signatures, especially in the maintenance of genomic stability [58]. In addition, cancer cells from adrenal cortex or colon tissue may have disparate circadian rhythms [59], which DNA damage repair may occur at different time point and then need different DDR pathways to repair the DNA damage [60, 61]. BMAL1 is a multidomain protein which can form different protein complex in different surrounding environment and thus may play different roles in DNA damage repair. In our study, knockdown of BMAL1 impairs the HR-mediated DSBs repair and increases the DNA damage, whereas, Zeng et al. found that depletion of BMAL1 decreases the DNA damage induced by VP-16. This may explain why BMAL1 has distinct roles in tumor proliferation and chemoresistance. This phenomenon has been verified by lots of proteins, such as p21, which has been found to promote cisplatin-induced cell death in glioma [62] and protect colon cancer against doxorubicin-induced apoptosis [63].

Due to the Bmal1 has dual role in cancer therapy, there is a need to develop those selective BMAL1 agents that promote only its tumor suppressor activity. Therefore, additional studies are warranted to explore the basic role of BMAL1 in cancer and its mechanism of action in the development and treatment of cancer.

## Materials and methods

### Cell culture and transfection

HEK293T, U2OS and SW-13 cells were obtained from American Type Culture Collection. U2OS cells with DR‐GFP or EJ5-GFP integration were kindly provided by Zhou Songyang at the School of Life Science, Sun Yat-sen University. All cells were grown in DMEM (GIBCO) with 10% fetal bovine serum (GIBCO) and 100 U/ml penicillin/streptomycin (GIBCO). Cells were cultured at 37 °C and 5% CO2. All the cells were verified by standardized short tandem repeat analysis. All cell lines were negative for mycoplasma contamination.

Plasmids were transfected into indicated cells using Lipo3000 following the manufacturer’s instruction (Thermo Fisher Scientific). siRNAs were transfected into indicated cells using Lipofectamine RNAiMAX reagent (Thermo Fisher Scientific). siRNA sequences: BMAL1-1: 5′-GACCCUCAUGGAAGGUUAGAAUAUA-3′; BMAL1-2: 5′- CAACUACAGCCAGAAUGAUCUGAUU-3′; shRNA sequences: BMAL1-1: 5′-AGAACCCAGGTTATCCATATT-3′; BMAL1-2: 5′- TAGGCACATCGTGTTA-3′; CLOCK-1: 5′-GCGAGGAACAATAGACCCAAA-3′; CLOCK-2: 5′-CGACGAGAACTTGGCATTGAA-3′.

### Plasmids

Wild-type BMAL1 (BMAL1-WT) and CLOCK (CLOCK-WT) genes were amplified from MRC5 mRNA and cloned into pCDNA3.1-Flag. The BMAL1 S183A (BMAL1-S183A) was generated by site-directed mutation based on wild-type BMAL1. The reduced acetyltransferase activity CLOCK (CLOCK-MUT) was generated by site-directed mutation based on wild-type CLOCK [44]. The shRNA-resistant CLOCK was generated by synonymously mutating the sequences at shRNA targeting site based on wild-type CLOCK. Plasmid pBABe-HA-ER-I-PpoI and pCBASceI were purchased from Addgene (Plasmid #32565 and #26477).

### Cell treatment

Unless otherwise indicated, cell lines were treated with Zeocin (100 μg/mL, Thermo Fisher) for 4 h; B02 (27.4 μM, Selleck) or NU7441 (250 nM, Selleck) or KU60019 (10 μM, Selleck) or VE-821 (10 μM, Selleck) for 24 h; 4-hydroxytamoxifen (1 μM, MCE) for 16 h; VP16 (5 mg/kg, MCE); Cisplatin (3 μM, MCE) for 4 h; CPT (100 μM, MCE) for 1 h; H_2_O_2_ (10 μM, ZHIYUAN) for 1 h, UVC (50 J/m^2^ and released 5 min at 37 °C).

### Immunofluorescence

For immunofluorescence experiments, cells plated on coverslips were pre-permeabilized with 0.5% Triton X-100 at 4 °C for 1 min followed by fixation in 4% paraformaldehyde once and permeabilized with 0.1% Triton at RT for 30 min. Cells were blocked with 5% BSA in PBS and incubated sequentially with primary antibody and fluorescence-labeled secondary antibody. For BrdU staining, U2OS cells transfected with siBMAL1 or overexpressed with BMAL1-WT or BMAL1-S183A were labeled with BrdU for 24 h and treated with Zeocin for 4 h. Then the cells were pre-permeabilized with 0.5% Triton X-100 at 4 °C for 5 min followed by fixation in 4% paraformaldehyde once. Cells were blocked with 5% BSA in PBS and incubated sequentially with BrdU antibody and fluorescence-labeled secondary antibody. Coverslips mounted with Vectashield mounting medium containing DAPI (Vector Laboratories) were visualized and analyzed using fluorescence microscopy. Antibodies used: BMAL1 (1:100, NB100-2288, Novus Biologicals), CLOCK (1:100, NBP1-51610, Novus Biologicals), mouse-γH2AX (1:400, ab26350, Abcam), rabbit-γH2AX (1:400, 9718, CST), RPA1 (1:100, sc-28304, Santa Cruz), RAD51 (1:400, ab133534, Abcam), BrdU (1:300, 347580, BDIS), Flag (1:1000, M20008, Abmart).

### Comet assay

Cells knockdown of BMAL1 were harvested and mixed with 0.5% low melting temperature agarose and layered on slides pre-coated by 1.5% normal agarose. Slides were lysed in 2.5 M NaCl, 100 mM EDTA, 10 mM Tris (pH 8.0), 0.5% Triton X-100, 3% DMSO, 1% N-lauroylsarcosine overnight at 4 °C and then electrophoresis in 300 mM sodium acetate, 100 mM Tris-HCl, 1% DMSO at 1.5 V/cm for 20 min. After neutralization with 0.4 M Tris-HCl (pH 7.3), slides were washed and dried with ethanol. The slides were then mounted with Vectashield mounting medium containing DAPI (Vector Laboratories) and visualized under fluorescence microscopy. Analysis was performed with CASP.

### Constant-field gel electrophoresis

Constant-field gel electrophoresis (CFGE) assay was performed as described previously [64]. Briefly, Cells knockdown of BMAL1 or CLOCK were harvested and imbedded in 0.7% agarose, lysed with 0.5% SDS in Tris-HCl (pH 7.4) and digested with RNase A (100 μg/ml) and proteinase K (250 μg/ml) at 37 °C overnight. Gel electrophoresis was performed using 0.7% agarose in TAE buffer. Genomic DNA was stained with gel-red.

### Chromatin immunoprecipitation

Chromatin immunoprecipitation (ChIP) was performed as previously described [22]. HEK293T cells were co-transfected with pBABe-HA-ER-I-PpoI and siBMAL1 (siBMAL1-1, siBMAL1-2) or shCLOCK (shCLOCK-1, shCLOCK-2) or BMAL1-WT or BMAL1-S183A or CLOCK and treated with 1 μM 4-hydroxy-Tamoxifen (4-OHT) for 16 h. Cells were then collected for ChIP. The pull-down efficiency was calculated as “% of input” after subtracting background signal. Anti-Flag beads (HY-K0207-1mL, MCE), antibodies to acetylated histone H4 (06-866, Millipore) or IgG (D110502, Sangon Biotech) were used. qPCR primers for elution detection were as follows: 28sDNA-L-F: 5′- GGGGAATCCGACTGTTTA-3′; 28sDNA-L-R: 5′-ACTGGGCAGAAATCACAT-3′; 28sDNA-R-F: 5′-TGGAGCAGAAGGGCAAAAGC-3′; 28sDNA-R-R: 5′-TAGGAAGAGCCGACATCGAAGG-3′; DAB1-L-F: 5′-CCCTCTCACTTTGGAGGGGAC-3′; DAB1-L-R: 5′-TTTATATAAGATGCCTGCCTGC-3′; DAB1-R-F: 5′-TGTGCTCTTTCCACTGTGGT-3′; DAB1-R-R: 5′-ATCACACTCTGCCACGTATG-3′.

### RNA purification and RT-qPCR

RNA was purified using RNAiso Plus (Takara) according to the manufacturer’s protocol. cDNA was generated from 1 μg of purified RNA using a TransScript® One-Step gDNA Removal and cDNA Synthesis SuperMix (TransGen Biotech, AT311) according to the manufacturer’s protocol. One microliter out of 20 μl cDNA was used to perform qPCR using the RealStar Power SYBR Mixture (GenStar) with the following gene specific primers: GAPDH-F: 5′-AGCCACATCGCTCAGACAC-3′; GAPDH-R: 5′-GCCCAATACGACCAAATCC-3′; PER1-F: 5′-GCCAACCAGGAATACTACCAGC-3′; PER1-R:5′-GTGTGTACTCAG-ACGTGATGTG-3′; PER2-F: 5′-GACATGAGACCAACGAAAACTGC-3′; PER2-R: 5′-AGGCTAAAGGTATCTGGACTCTG-3′; NR1D2-F: 5′- TTTAGTGGCATGGTTCTACTGTG-3′; NR1D2-R: 5′- AGCCTTCGCAAGCATGAACT-3′.

### DNA end-resection assay

The level of ssDNA was quantitated by using the HA-ER-I-PpoI system. HEK293T cells were co-transfected with pBABe-HA-ER-I-PpoI and siBMAL1 or shCLOCK (shCLOCK-1, shCLOCK-2). Upon addition of 4-OHT, the I-PpoI enzyme localizes to the nucleus and generates DSBs at sequence specific I-PpoI sites (TAACTATGACTCTCTTAAGGTAGCCAAAT-3), such as 28S rDNA. Genomic DNA was extracted and digested with DSN (4 units for 1 μg DNA) at 37°C 2 h. After digestion, the ssDNA at the specific I-PpoI cutting sites in 28S rDNA was purified by phenol-chloroform extraction and measured by qPCR with the following primers sets: 28S rDNA-0.1-F: 5′-CGAGATTCCCACTGTCCCTAC-3′; 28S rDNA-0.1-R: 5′-TCTTCTTTCCCCGCTGATTCC-3′; 28S rDNA-0.5-F: 5′-GTACACCTGTCAAACGGTAAC-3′; 28S rDNA-0.5-R: 5′-TTTTGCCCTTCTGCTCCA-3′; 28S rDNA-1-F: 5′-TTTGGTGTATGTGCTTGG-3′; 28S rDNA-1-R: 5′-TTAGAGGCGTTCAGTCAT-3′.

### Determination of frequency of HR or NHEJ

A U2OS cell clone stably expressing HR reporter DR‐GFP or EJ5-GFP was described previously [33, 34]. Expression of I‐SceI endonuclease will generate a site‐specific DSB between the mutated GFP genes, which when repaired by gene conversion, results in a functional GFP gene. Briefly, 0.2 × 10^6^ U2OS DR‐GFP or EJ5-GFP cells were plated onto 6-well dishes and transfected pCBASceI and BMAL1-WT, BMAL1-S183A or siBMAL1 (siBMAL1-1, siBMAL1-2) using lipo3000 or RNAiMAX following the manufacturer’s instruction. The cells were harvested 2 days after transfection and subjected to flow cytometry analysis to determine percentages of GFP‐positive cells, which result from HR or NHEJ repair induced by DNA DSBs. Means were obtained from three independent experiments.

### Cell treatment and cell viability

SW-13 cells were transferred indicated plasmids. Cells were treated with Zeocin at indicated doses for 2 days and subjected to viability assay. Cell viability was measured with CCK-8 kit (Bimake) following the protocol provided by manufactory.

### Establishment of xenograft models

A total of 1.0 × 10^7^ normal (shNC) or BMAL1-depleted (shBMAL1) SW-13 cells were inoculated subcutaneously into the dorsal flank of the 4-week-old female BALB/c nude mice in 100 μl PBS. shNC or shBMAL1 SW-13 cells bearing mice were randomly divided into control and VP16 treatment groups. 5 mg/kg of VP16 was injected (Intraperitoneal injection) every other day for 8 days. No animal died during the experimental period. All experiments were approved by Sun Yat-sen University Animal Care and Use Committee and were conducted in accordance with the National Institutes of Health (NIH) Guidelines for the Care and Use of Laboratory Animals. The tumor size was estimated from the measurements of the longest diameter across the tumor and the corresponding perpendicular diameter.

### Immunohistochemical analysis

shNC or shBMAL1 Tumors were fixed overnight in 4% paraformaldehyde at room temperature, transferred to 70% ethanol and embedded in paraffin. 4μm sections on slides were dewaxed in xylene, and then sequentially rehydrated in 100%, 95%, 70% ethanol and PBS buffer. For γH2AX immunohistochemistry, sections were blocked with 5% goat serum in PBS buffer, then incubated overnight with primary antibodies to γH2AX at 4 °C, washed three times with 1×PBS, and incubated with secondary antibodies. Sections were washed three times with 1×PBS and stained with DAB and hematoxylin (for DNA staining) according to standard protocols. Primary antibody used: γH2AX (1:400, 9718, CST). Second antibody used: HRP-conjugated anti-rabbit (KPL, Inc). To assess the frequency of apoptosis, tissue was analyzed using a TdT-mediated dUTP-digoxigenin nick end labeling (TUNEL) method. Staining was performed according to manufacturer’s instructions (TUNEL assay Kit, C1086, Beyotime).

### Immunoblotting

Proteins were separated with SDS-PAGE and transferred to PVDF membrane. The following antibodies for immunoblotting: BMAL1 (1:2000, NB100-2288, Novus Biologicals), CLOCK (1:2000, NBP1-51610, Novus Biologicals), γH2AX (1:2000, 9718, CST), ATM pS1981 (1:5000, ab81292, Abcam), ATM (1:5000, ab32420, Abcam), RAD51 (1:4000, ab133534, Abcam), ATR (1:5000, ab2905, Abcam), ATR pT1989 (1:1000, GTX128145, GeneTex), RPA1 (1:1000, sc-28304, Santa Cruz), Flag (1:1000, D191041, Sangon Biotech), GAPDH (1:5000, 60004-1-Ig, Proteintech), HRP-conjugated anti-rabbit or anti-mouse (KPL, Inc) were used as secondary antibody.

### Gene expression analysis

RNA-seq data about BMAL1 expression level and pathological stages of the patients suffering from Adrenocortical carcinoma (ACC) were obtained from the TCGA data portal (https://tcga-data.nci.nih.gov/tcga). These patients were divided into four groups according to clinical pathological stages (Stage I-IV).

### Kaplan–Meier survival analysis

BMAL1 expression level of patients were from TCGA data portal (https://tcga-data.nci.nih.gov/tcga). Corresponding survival data were from R package “survival” (https://CRAN.R-project.org/package=survival). Kaplan–Meier survival analysis was performed on patient’s BMAL1 expression data and survival data. A log-rank test was applied to compare the survival distributions of two groups: patients with relatively high BMAL1 expression and those with relatively low expression based on median of all acquired expression data. Survival curves were plotted by an R package “survminer” (http://www.sthda.com/english/rpkgs/survminer).

### Quantification and statistical analysis

GraphPad Prism 8 was used for statistical analysis. Results are shown as mean ± SEM and the unpaired Student’s two-tailed *t*-test was used to determine the statistical significance (^*^P < 0.05; ^**^P < 0.01; ^***^P < 0.001; ^****^P < 0.0001).

## Supplementary information

supplementary figure

## Data Availability

All relevant data are available from the authors upon request.
