# Supplementary material for: BMAL1 collaborates with CLOCK to directly promote DNA double-strand break repair and tumor chemoresistance
Source: Oncogene. 2023 Feb 2;42(13):967–79. doi: 10.1038/s41388-023-02603-y (PMC10038804; doi:10.1038/s41388-023-02603-y)
Supplement: Supplementary file 1 — supplementary figure [file 41388_2023_2603_MOESM1_ESM.pdf]

Supplementary Fig.1

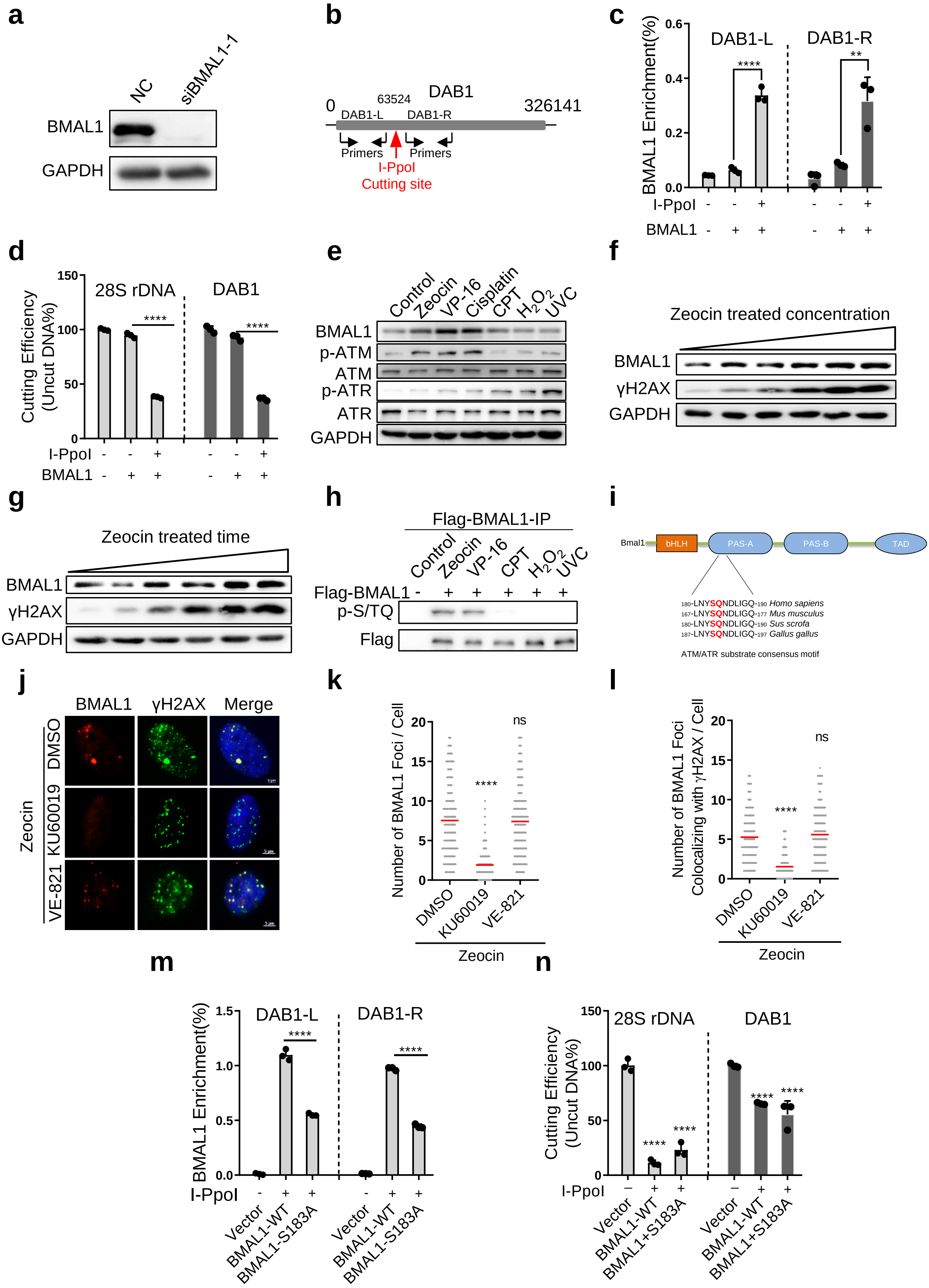

Supplementary Fig.1 Detection of BMAL1 at DSB sites.

(**a**) Immunoblotting analysis the knocking down efficiency of BMAL1. U2OS cells were transfected with NC or siBMAL1-1.

(**b**) Location of I-PpoI cutting site at DAB1. The positions of primers used for ChIP-qPCR are indicated by arrows.

(**c**) ChIP-qPCR for DAB1 with or without cutting by I-PpoI and binding by BMAL1.

(**d**) Detection of cleavage efficiency of I-PpoI at 28S rDNA and DAB1. qPCR was performed for I-PpoI transfected HEK293T cells with over-expression of Vector, or BMAL1. I-PpoI non-transfected cells were used as a control.

(**e**) Immunoblot analysis of BMAL1, activated ATM, total ATM, activated ATR and total ATR in U2OS cells treated with Zeocin, VP16, Cisplatin, CPT, H_2O_2 or UVC.

(**f**) Immunoblot analysis of BMAL1 and γH2AX in U2OS cell treated with 0, 25, 50, 100, 200, 400μg/mL Zeocin for 4 hours.

(**g**) Immunoblot analysis of BMAL1 and γH2AX in U2OS cell treated with 100μg/mL Zeocin for 0, 0.5, 1, 2, 4, 8 hours.

(**h**) Flag-BMAL1-overexpressing HEK293T cells were treated with Zeocin, VP16, CPT, H_2O_2 or UVC followed by pulldown with anti-Flag beads and immunoblotting with antibodies against p-S/TQ, Flag and γH2AX.

(**i**) Consensus ATM phosphorylation site on human BMAL1 (S183) and alignment with the conserved site on BMAL1 from other species.

(**j**) Detection of BMAL1 and γH2AX foci in U2OS cells that were incubated with KU60019 (10μM) or VE-821 (10μM) for 24h, followed by Zeocin treatment. Scale bar: 5μm.

(**k**) Quantification of panel **j**. The number of BMAL1 foci per cell (n≥100).

(**l**) Quantification of panel **j**. The number of BMAL1 foci colocalized with γH2AX foci.

(**m**) ChIP-qPCR for DAB1 with or without cutting by I-PpoI and binding by BMAL1-WT or BMAL1-S183A.

(**n**) Detection of cleavage efficiency of I-PpoI at 28S rDNA and DAB1. qPCR was performed for I-PpoI transfected HEK293T cells with over-expression of Vector, BMAL1-WT or BMAL1-S183A. I-PpoI non-transfected cells were used as a control.

All values are the average ±SEM of three independent experiments. Student's unpaired two-tailed t-test was used to determine the statistical significance (**P<0.01, ****P<0.0001).

Supplementary Fig.2

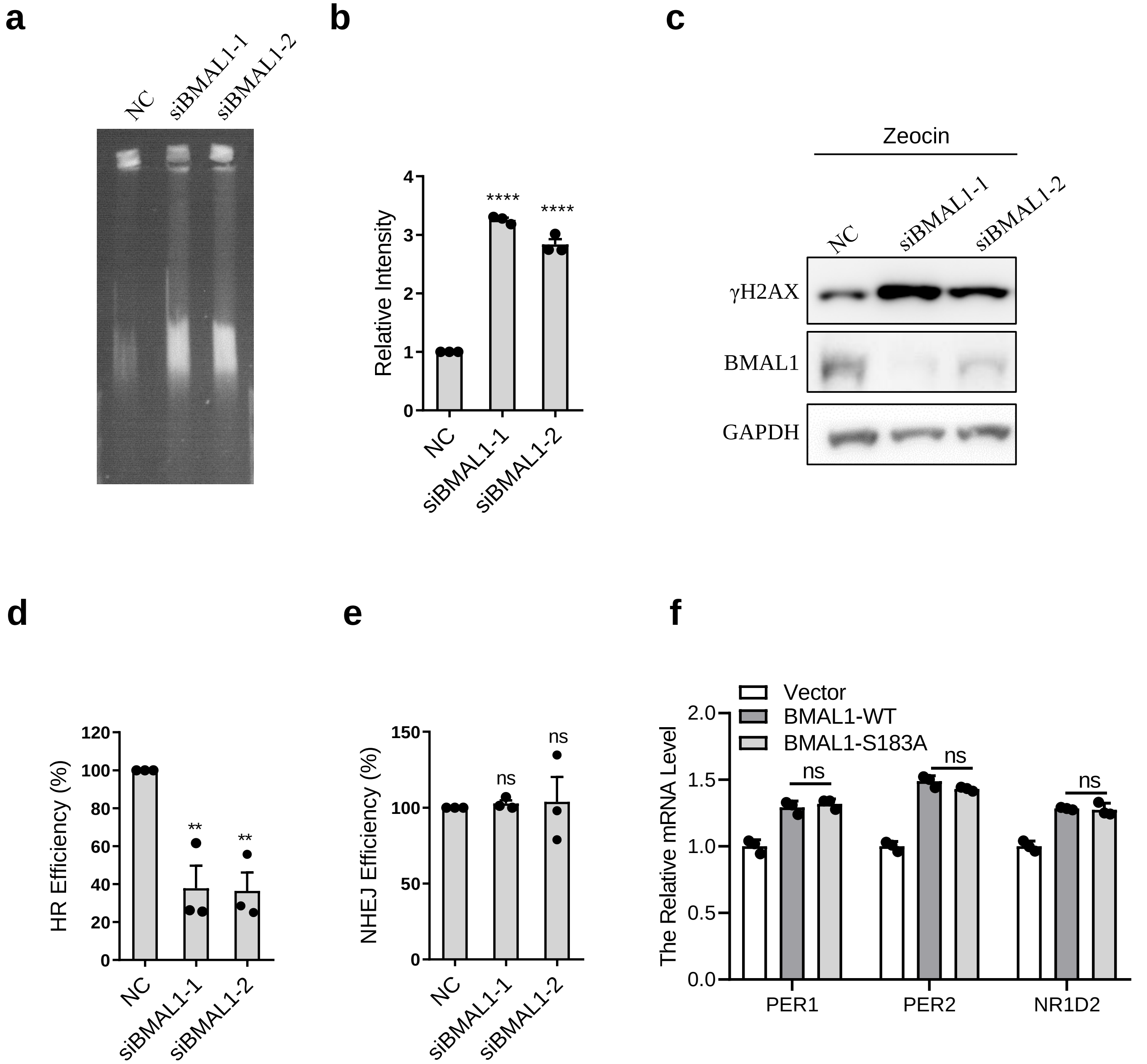

Supplementary Fig.2 BMAL1 is essential for HR-mediated DSBR.

(**a**) Constant-field gel electrophoresis (CFGE) analysis of DNA fragment released by DSBs on genome in U2OS cells transfected with NC, siBMAL1-1 or siBMAL1-2. Gel was stained with GelRed.

(**b**) Quantification of **a**. The relative amount of DNA fragments was determined by normalizing to the smear signal of cells transfected with NC.

(**c**) Immunoblot analysis of γH2AX and BMAL1. HEK293T cells transfected with NC, siBMAL1-1 or siBMAL1-2 were treated with Zeocin and incubated in fresh culturing medium for 6h.

(**d**) FACS analysis the frequency of HR in BMAL1 depleted U2OS DR-GFP cell line transfected with I-SceI.

(**e**) FACS analysis the frequency of NHEJ in BMAL1 depleted U2OS EJ5-GFP cell line transfected with I-SceI.

(**f**) RT-qPCR to determine the transcription level of BMAL1 downstream genes. HEK293T cells over-expressed with BMAL1-WT or BMAL1-S183A.

All values are the average ±SEM of three independent experiments. Student’s unpaired two-tailed t-test was used to determine the statistical significance (**$P<0.01$, ****$P<0.0001$).

Supplementary Fig.3

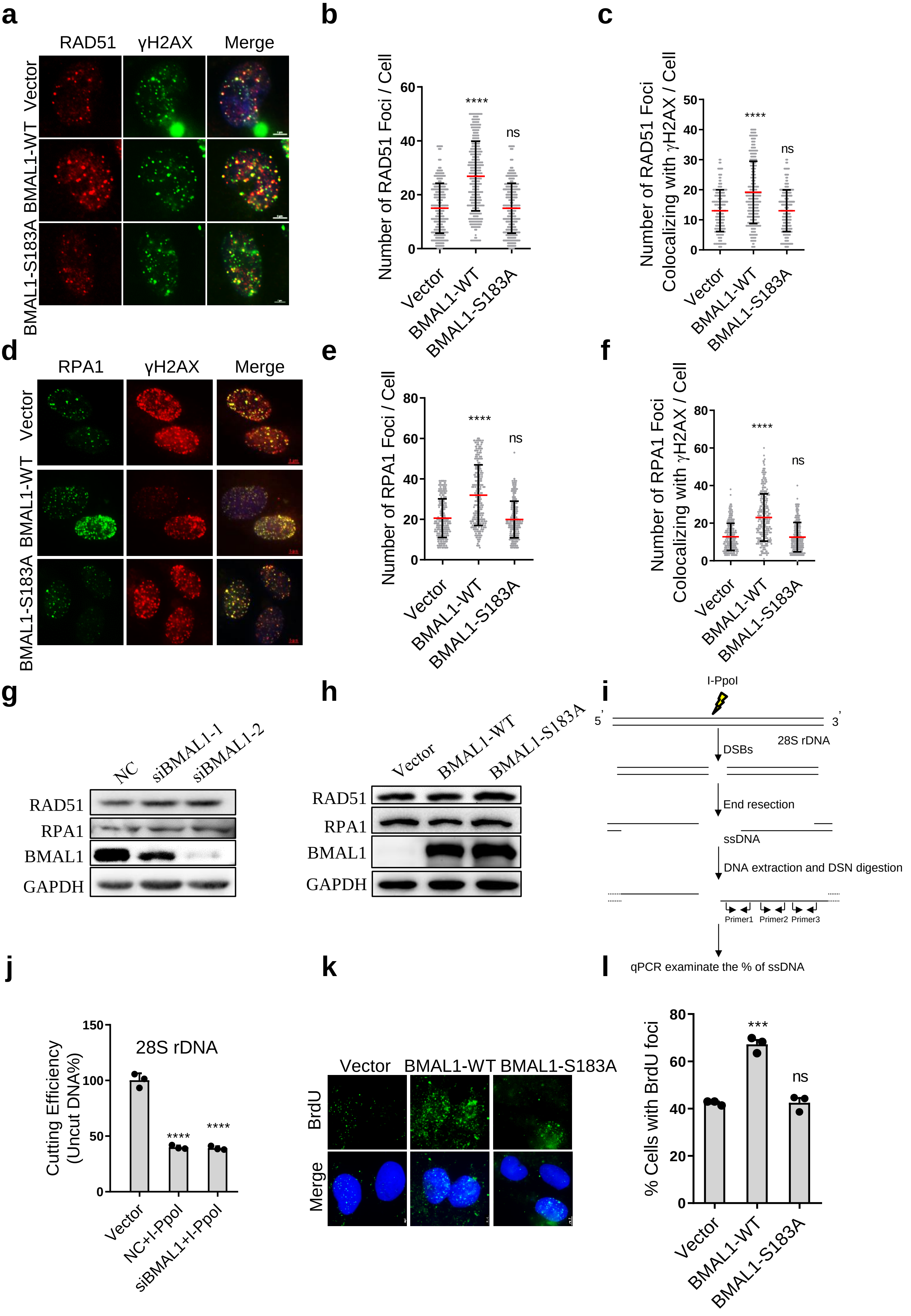

Supplementary Fig.3 BMAL1 facilitates the recruitment of RAD51 to DSB sites.

(**a**) Immunofluorescence detection of RAD51 and γH2AX foci in U2OS cells overexpressed with Vector, BMAL1-WT or BMAL1-S183A. Cells were treated with Zeocin. Scale bar: 5μm.

(**b**) Quantification of panel **a**. The number of RAD51 foci per cell (n≥100).

(**c**) Quantification of panel **a**. The number of RAD51 foci colocalized with γH2AX foci.

(**d**) Immunofluorescence detection of RPA1 and γH2AX foci in U2OS cells overexpressed with Vector, BMAL1-WT or BMAL1-S183A. Cells were treated with Zeocin. Scale bar: 5μm.

(**e**) Quantification of panel **d**. The number of RPA1 foci per cell (n≥100).

(**f**) Quantification of panel **d**. The number of RPA1 foci colocalized with γH2AX foci.

(**g**) Immunoblot analysis of RAD51, RPA1 and BMAL1 in U2OS cells transfected with NC, siBMAL1-1 or siBMAL1-2.

(**h**) Immunoblot analysis of RAD51, RPA1 and BMAL1 in U2OS cells overexpressed with Vector, BMAL1-WT or BMAL1-S183A.

(**i**) Schematic representation of the I-PpoI-based DNA end-resection assay in HEK293T cells.

(**j**) Detection of cleavage efficiency of I-PpoI at 28S rDNA. qPCR was performed for I-PpoI transfected HEK293T cells with knockdown of BMAL1. I-PpoI non-transfected cells were used as a control.

(k) Immunofluorescence detection of BrdU foci in U2OS cells overexpressed with Vector, BMAL1-WT or BMAL1-S183A. Cells were treated with Zeocin. Scale bar: 5μm.

(l) Quantification of panel k. The percentage of cell with BrdU foci (n≥100).

All values are the average ±SEM of three independent experiments. Student's unpaired two-tailed t-test was used to determine the statistical significance (***P< 0.001, ****P<0.0001).

Supplementary Fig.4

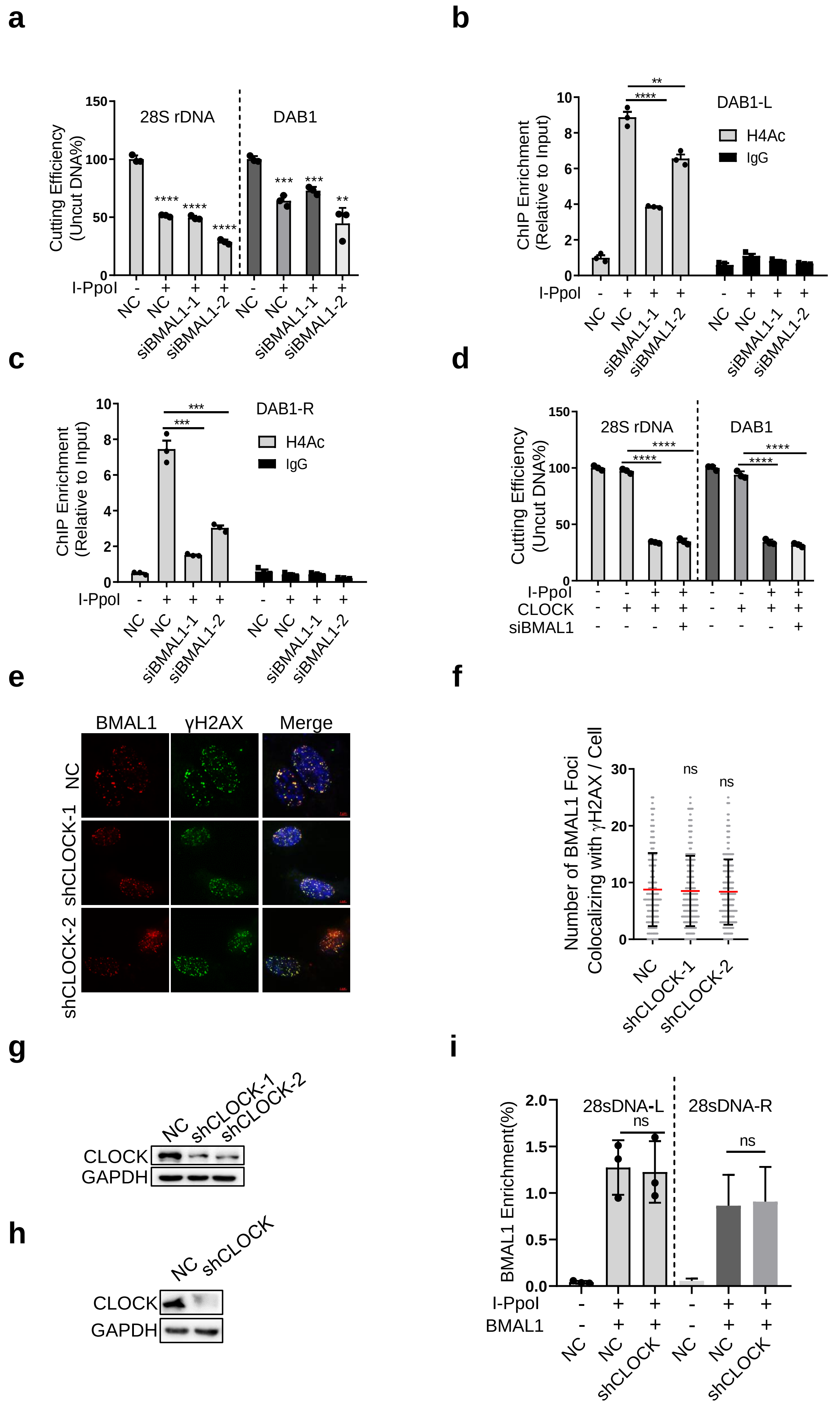

Supplementary Fig.4 BMAL1 is essential to the localization of CLOCK to DSB sites.

(**a**) Detection of cleavage efficiency of I-PpoI at 28S rDNA and DAB1. qPCR was performed for I-PpoI overexpressed HEK293T cells with or without knockdown of BMAL1 (NC, siBMAL1-1 or siBMAL1-2). I-PpoI non-transfected cells were used as a control.

(**b**)-(**c**) ChIP-qPCR detected the relative enrichment of Histone H4 acetylation (H4Ac) at DAB1 with or without cutting by I-PpoI. HEK293T cells were transfected with NC, siBMAL1-1 or siBMAL1-2.

(**d**) Detection of cleavage efficiency of I-PpoI at 28S rDNA and DAB1. qPCR was performed for I-PpoI overexpressed HEK293T cells with transfection of Vector, NC and Clock or siBMAL1 and Clock. I-PpoI non-transfected cells were used as a control.

(**e**) Immunofluorescence detection of BMAL1 and γH2AX foci in normal and CLOCK depleted U2OS (shCLOCK-1, shCLOCK-2). Cells were treated with Zeocin. Scale bar: 5μm.

(**f**) Quantification of panel **e**. The number of BMAL1 foci colocalized with γH2AX foci. ($n \geq 100$).

(**g**) Immunoblot analysis of CLOCK in normal and CLOCK stably depleted U2OS cells (shCLOCK-1, shCLOCK-2).

(**h**) Immunoblot analysis of CLOCK in normal and CLOCK stably depleted HEK293T cells.

(**i**) ChIP-qPCR showing the relative enrichment of BMAL1 at 28S rDNA with or without cutting by I-PpoI. HEK293T cells were transfected with NC or shCLOCK-2.

All values are the average ±SEM of three independent experiments. Student's unpaired two-tailed t-test was used to determine the statistical significance (**$P<0.01$, ***$P<0.001$, ****$P<0.0001$).

Supplementary Fig.5

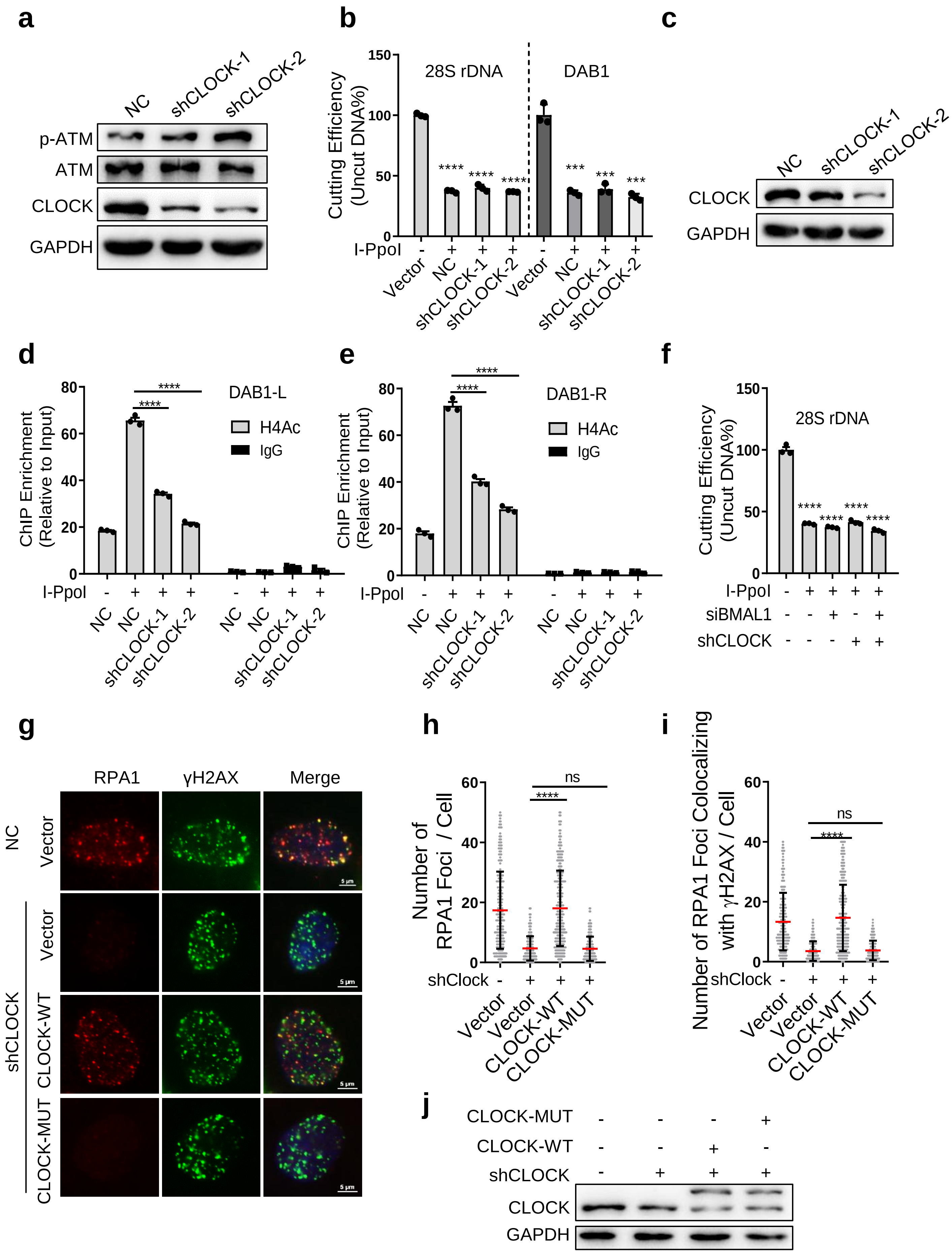

Supplementary Fig.5 H4Ac mediated by CLOCK is important for HR repair.

(**a**) Immunoblot analysis of phosphorylated ATM (p-ATM), total ATM and CLOCK in normal and CLOCK stably depleted U2OS cells (shCLOCK-1, shCLOCK-2).

(**b**) Detection of cleavage efficiency of I-PpoI at 28S rDNA and DAB1. qPCR was performed for I-PpoI overexpressed HEK293T cells transfected with NC, shCLOCK-1 or shCLOCK-2. I-PpoI non-transfected cells were used as a control.

(**c**) Immunoblot analysis of CLOCK in normal and CLOCK stably depleted HEK293T cells (shCLOCK-1, shCLOCK-2).

(**d**)-(**e**) ChIP-qPCR showing the relative enrichment of Histone H4 acetylation (H4Ac) at DAB1 with or without cutting by I-PpoI. HEK293T cells were transfected with shNC, shCLOCK-1 or shCLOCK-2.

(**f**) Detection of cleavage efficiency of I-PpoI at 28S rDNA. qPCR was performed for I-PpoI overexpressed HEK293T cells transfected with indicated siRNAs or shRNAs. I-PpoI non-transfected cells were used as a control.

(g) Ectopic expression of shCLOCK-resistant CLOCK-WT, but not shCLOCK-resistant CLOCK-MUT, rescues RPA1 foci at DSBs in CLOCK-deficient U2OS cells. Cells were treated with Zeocin and collected for detection of RPA1 and γH2AX. Scale bar: 5μm.

(h) Quantification of panel **g**. The number of RPA1 foci per cell (n≥100).

(i) Quantification of panel **g**. The number of RPA1 foci colocalized with γH2AX foci.

(j) Immunoblot analysis of CLOCK in CLOCK stably depleted U2OS cells transfected with Vector, shCLOCK-resistant CLOCK-WT or shCLOCK-resistant CLOCK-MUT. Cells were treated with Zeocin and collected for immunoblotting with indicated antibody.

All values are the average ±SEM of three independent experiments. Student's unpaired two-tailed t-test was used to determine the statistical significance (***$P<0.001$, ****$P<0.0001$).

Supplementary Fig.6

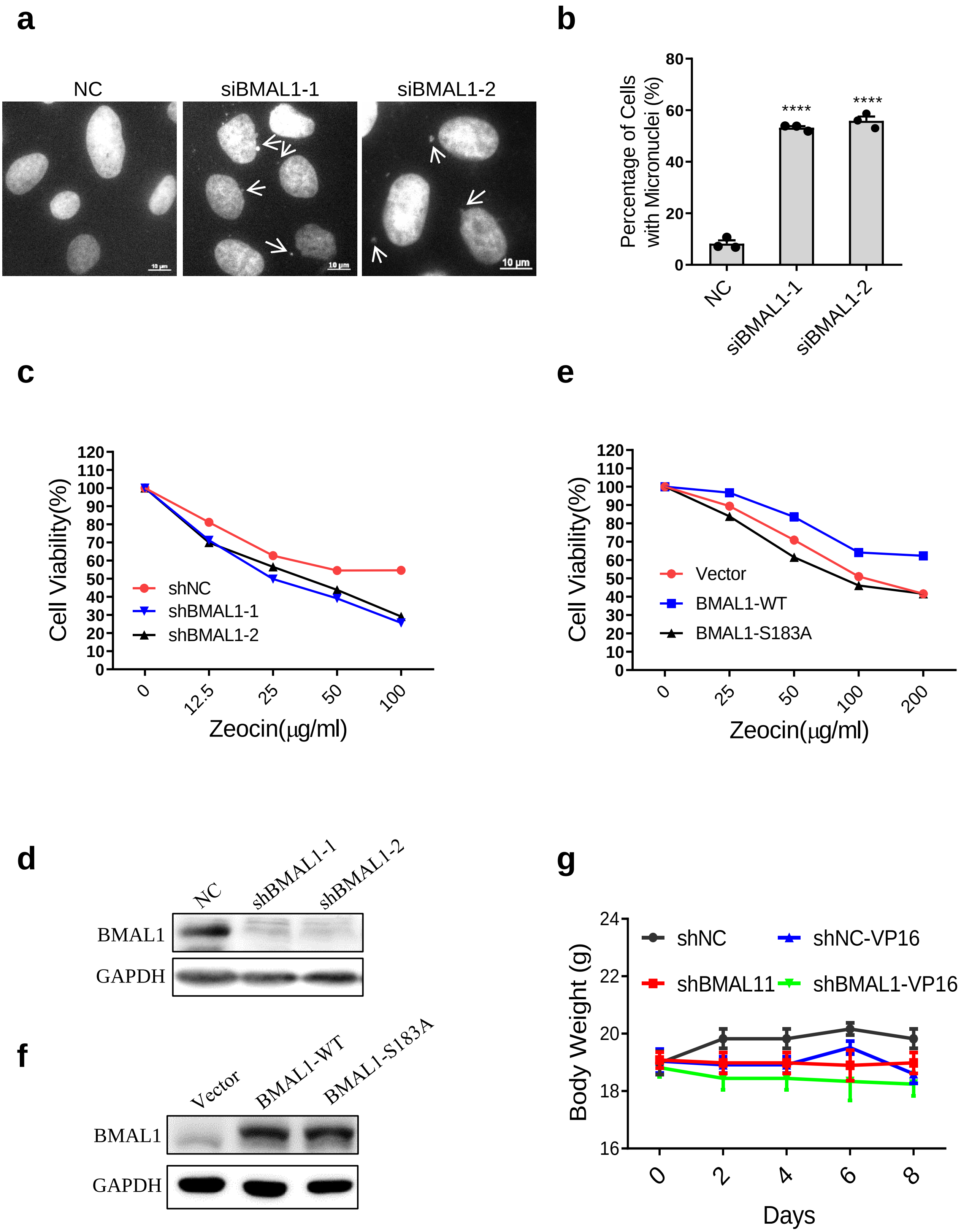

Supplementary Fig.6 BMAL1 is important for genomic stability and cell survival upon DNA damage agents.

(**a**) Representative image showing micronuclei in U2OS cells transfected with NC, siBMAL1-1 or siBMAL1-2. Cells were treated with Zeocin before analysis. Scale bar: 10μm.

(**b**) Quantification of **a**. The percentage of cells with micronuclei ((n≥100 cells).

(**c**) Cell viability analysis of normal and BMAL1 depleted (shBMAL1-1, shBMAL1-2) SW-13 cells that were treated with increasing concentrations of Zeocin.

(**d**) Immunoblot analysis of BMAL1 in SW-13 cells transfected with NC, shBMAL1-1 or shBMAL1-2.

(**e**) Cell viability analysis of Vector, BMAL1-WT or BMAL1-S183A overexpressed SW-13 cells that were treated with increasing concentrations of Zeocin.

(**f**) Immunoblot analysis of BMAL1 in SW-13 cells overexpressed Vector, BMAL1-WT or BMAL1-S183A.

(**g**) The body weight of the mice was measured over the treatment period.

All values are the average ±SEM of three independent experiments. Student's unpaired two-tailed t-test was used to determine the statistical significance (****P<0.0001).
